# Local transmural action potential gradients are absent in the isolated, intact dog heart but present in the corresponding coronary‐perfused wedge

**DOI:** 10.14814/phy2.13251

**Published:** 2017-05-29

**Authors:** Bastiaan J. Boukens, Veronique M. F. Meijborg, Charly N. Belterman, Tobias Opthof, Michiel J. Janse, Richard B. Schuessler, Ruben Coronel, Igor R. Efimov

**Affiliations:** ^1^ Department of Biomedical Engineering George Washington University Washington District of Columbia; ^2^ Department of Medical Biology University of Amsterdam Amsterdam The Netherlands; ^3^ Department of Experimental and Clinical Cardiology University of Amsterdam Amsterdam The Netherlands; ^4^ Netherlands Heart Institute Holland Heart House Utrecht The Netherlands; ^5^ Institut LIRYC Electrophysiology and Heart Modeling Institute fondation Bordeaux Université Pessac‐ Bordeaux France; ^6^ Department of Medical Physiology University of Utrecht Utrecht The Netherlands; ^7^ Department of Surgery Washington University St Louis Missouri

**Keywords:** APD, ARI, Repolarization, Wedge and Intact heart

## Abstract

The left ventricular (LV) coronary‐perfused canine wedge preparation is a model commonly used for studying cardiac repolarization. In wedge studies, transmembrane potentials typically are recorded; whereas, extracellular electrical recordings are commonly used in intact hearts. We compared electrically measured activation recovery interval (ARI) patterns in the intact heart with those recorded at the same location in the LV wedge preparation. We also compared electrically recorded and optically obtained ARIs in the LV wedge preparation. Five Langendorff‐perfused canine hearts were paced from the right atrium. Local activation and repolarization times were measured with eight transmural needle electrodes. Subsequently, left ventricular coronary‐perfused wedge preparations were prepared from these hearts while the electrodes remained in place. Three electrodes remained at identical positions as in the intact heart. Both electrograms and optical action potentials were recorded (pacing cycle length 400–4000 msec) and activation and repolarization patterns were analyzed. ARIs found in the subepicardium were shorter than in the subendocardium in the LV wedge preparation but not in the intact heart. The transmural ARI gradient recorded at the cut surface of the wedge was not different from that recorded internally. ARIs recorded internally and at the cut surface in the LV wedge preparation, both correlated with optically recorded action potentials. ARI and RT gradients in the LV wedge preparation differed from those in the intact canine heart, implying that those observations in human LV wedge preparations also should be extrapolated to the intact human heart with caution.

## Introduction

Perfused ventricular wedge preparations are frequently used in (patho)physiological studies, because they allow assessment of transmural electrophysiological parameters with high spatial resolution, particularly when optical methods are applied (Di Diego et al. 2013). The preparation has been often used in, for example, the study of ectopic beats in heart failure (Lang et al. 2015) and in the study of the long QT syndrome (Shimizu and Antzelevitch 1998). It has not been established whether results obtained in the experimental model wedge preparation are in agreement with those found in the intact heart (Opthof et al. 2007a). In addition, optical methods are often used in studies in wedge preparations because they provide high spatial resolution. The use of optical methods in blood‐perfused intact hearts of large mammals is hampered by high absorption of excitation light by hemoglobin.

The debate on the validity of wedge preparations for cardiac electrophysiology has been fueled by the question of whether mid‐myocardial (Mid) repolarization characteristics are relevant for the genesis of the T wave or for arrhythmogenesis (Poelzing and Rosenbaum 2004; Opthof et al. 2009; Patel et al. 2009). Yan et al. (1998) reported that the left ventricular (LV) wedge preparation obtained from the canine heart harbored a Mid layer of myocytes (“M cells”) with an extremely long action potential. Microelectrode recordings in transmural shavings from the LV wall previously had suggested their existence (Sicouri and Antzelevitch 1991). It was also hypothesized that transmural heterogeneity in repolarization gives rise to the T wave observed in the electrocardiogram (ECG) of the intact heart. However, subsequent studies in the intact canine heart with transmural needle electrodes could not confirm extremely long activation recovery intervals (ARI, index of action potential duration [APD]) (Opthof et al. 2007b) or long refractory periods (Voss et al. 2009) in the Mid of the LV wall. The discrepancy between observations in the LV wedge and the intact heart centered on the validity of the model (wedge vs. intact heart, discussed at length by Opthof et al. (2007a) as well as on the validity of the methods (optical vs. electrical mapping).

The question of why results obtained in the wedge preparation differ from those in the intact heart has remained unanswered. Although the relation between ARI and APD has been thoroughly investigated (Haws and Lux 1990; Coronel et al. 2006), the difference in experimental approaches between studies performed in the wedge preparation (i.e., APDs, often measured with optical dyes or microelectrodes) and the intact heart (i.e. ARIs, measured with extracellular plunge needle electrodes) left room for differing explanations. This study aims to answer the following questions: (1) do ARIs recorded in the wedge preparation correlate with optically recorded APDs in the wedge preparation?; and (2) do ARIs recorded from the wedge preparation correlate with ARIs recorded from the intact heart?

## Methods

The experimental protocol complied with the Guide for the Care and Use of Laboratory Animals (US National Institutes of Health Publication 85–23, revised 1996), and was approved by the Institutional Review Board of Washington University School of Medicine and by the Washington University Animal Care and Use Committee.

### Excision of the Heart

Five normal mongrel canines (approximate weight, 25 kg) were anesthetized with intravenous propofol (5.5 mg/kg body weight) and deep anesthesia was maintained with pentobarbital (3 mg/kg/h) when necessary. The animals were intubated with an endotracheal tube and were maintained with isoflurane (2–3%). A mid‐sternal thoracotomy then was performed and heparin (5000 IU) (Leo Pharma) was injected intravenously. Tyrode's solution (in mmol/L: 128.2 NaCl, 4.7 KCl, 1.19 Na H_2_PO_4_, 1.05 MgCl_2_, 1.3 CaCl_2_, 27.0 NaHCO_3_, and 11.1 glucose) was infused while 1–2 L blood was collected. Cardioplegia solution (in mmol/L: 110 NaCl, 1.2 CaCl_2_, 16 KCl, 16 MgCl, 10 NaHCO_3_, and 9 glucose) was then injected into the aorta to arrest the heart. The heart was excised and submerged into cold cardioplegia solution.

### Langendorff preparation

After cannulation of the aorta, the excised heart was mounted to a Langendorff setup and retrogradely perfused with a mixture (1:2) of blood and Tyrode's solution (150 mL/min). Myocardial temperature was maintained at 37.0–37.5°C throughout the experiment and the pH of the oxygenated perfusate was maintained at 7.4. The stimulus electrode was positioned on the right atrium.

Transmural multi‐electrode (0.5 mm diameter) needles were inserted into the heart in a predefined pattern. In the anterior/lateral part of the LV, eight needles (four electrodes/needle, interelectrode distances = 4 mm) were inserted and connected to a 256‐channel amplifier (24 bit dynamic range, 122.07 nV LSB, total noise 0.5 *μ*V [BioSemi]). Signals were recorded at a sampling frequency of 2048 Hz (bandwidth [−3 dB] DC −400 Hz). The active common mode electrode was positioned in the aortic root. Stimulation pulse amplitude was 1.5 times the stimulation threshold and pulse width was 2 msec. Recordings were made during atrial pacing at cycle lengths of 800 msec.

### Wedge preparation

After the Langendorff experiment was concluded, ventricular fibrillation (VF) was induced with a 5 V battery and the heart was immersed into cold Tyrode's solution. A transmural wedge preparation was removed from the anterior‐lateral LV free wall after which VF stopped. The wedge was perfused via the circumflex artery. The stimulus electrode was located at the endocardium. The wedge preparation still contained the needle electrodes that were placed in the intact heart [3 needles, 12 electrodes]. The dimensions of the wedge preparation were ±3 × ±1.5 × ±1 cm. Tissue distal from the constriction of the arteries was trimmed and the wedge preparation was placed into the optical mapping setup and perfused at 37°C with Tyrode's solution (15 mL/min). The pH was maintained at 7.4 by equilibration with a mixture of 95% O_2_ and 5% CO_2_.

For recording of optical action potentials (OAP), the preparations were perfused with 10 *μ*Mol/L di‐4‐ANEPPS (Molecular Probes, Eugene, OR) for 10 min. The excitation‐contraction uncoupler blebbistatin (10 *μ*mol/L, Tocris Bioscience, Ellisville, MO) was added to the perfusate to remove motion artifacts. Excitation light was delivered by a 520 ± 5 nm light‐emitting diode (Prizmatix, Southfield, MI) and emitted fluorescence was filtered > 610 nm and recorded by a CMOS‐sensor (100 × 100 elements, 1 kHz, MICAM Ultima, SciMedia Ltd., Costa Mesa CA). A pseudo‐electrocardiogram was recorded by placing the electrode located at the epicardial side of the wedge into the positive input and the electrode located at the endocardial side into the negative input of the amplifier. The two electrodes were placed at the endo‐ and epicardial sides of the wedge preparation at a distance of 3 cm from endo‐ and epicardium, respectively (PowerLab 26T; AD‐Instruments, Colorado Springs, CO). Recordings were made during endocardial stimulation at intervals of 400, 500, 600, 800, 1000, and 4000 msec.

### Data analysis

Electrical and optical signal analysis was performed offline using custom‐made data analysis software based on MATLAB2014b (Mathworks Inc., Natick, MA) (Potse et al. 2002). The reference time in the recordings was defined as the start of the stimulus artifact (during ventricular pacing) or the earliest onset of the QRS complex (during atrial pacing). In the local unipolar electrograms, electrical activation times (AT) were defined as the interval between the reference time and the time of the maximum negative slope of the RS complex of the local electrograms. Repolarization times (RT) were defined as the interval between the reference time and the time of the maximum positive slope of the T wave of the local electrograms. ARIs were calculated by subtracting the local AT from the local RT. ARI variability was determined as the standard deviation of the difference between the ARI at an activation and the ARI at the subsequent activation. In the optical signals, the local AT was defined as the maximum positive dV/dt of the optical action potential (OAP) relative to the reference time. The RT was calculated as the interval between the moment of 80% of repolarization and the reference time (Potse et al. 2009). APD was derived from the subtraction of RT and AT.

### Statistics

Group comparisons were made using (three‐way and/or repeated) analysis of variance. Mauchly's Test of Sphericity indicated that the assumption of sphericity had not been violated. Values were shown as mean ± SEM. A *P* < 0.05 was considered statistically different.

## Results

### Repolarization patterns in the Langendorff‐perfused intact heart

**Figure 1:**
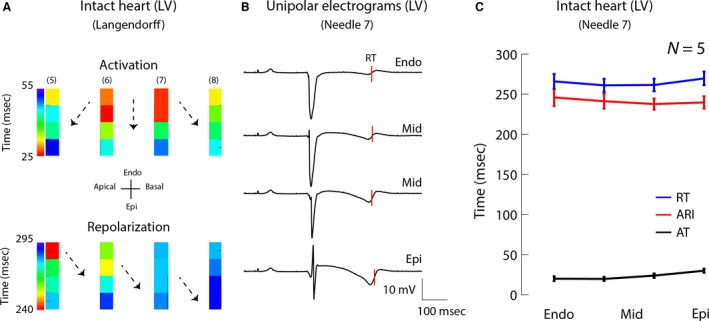
Activation and repolarization sequence in the left ventricle of a Langendorff‐perfused canine heart. (A) Activation (upper) and repolarization (lower) pattern in a section of the left ventricle based on four transmural needle electrodes (pacing interval 800 msec). Black dots represent electrodes. Areas between the black dots were interpolated. (B) Unipolar electrograms recorded with needle 7. The red lines indicate the moments of repolarization along needle 7. (C) The line graph shows the average activation and repolarization times and ARI gradient along needle 7 in five animals. LV, left ventricle; AT, activation time; RT, repolarization time; Endo, subendocardium; Mid, mid‐myocardium; Epi, subepicardium; ARI, activation recovery interval.

Heart rate was 103 ± 6 msec before isolation and in all dogs, the T waves were positive in all leads of the surface ECG with a QT interval of 256 ± 6 msec. In the Langendorff setup, the hearts were paced with a cycle length of 800 msec. Figure 1A shows a typical example of a reconstructed activation and repolarization pattern in one heart based on 16 sites from four transmural needle electrodes with four terminals each. The activation front started in the central endocardial region and propagated to the apex and base along the subendocardium and, as expected, from the subendocardium (Endo) to the subepicardium (Epi). All unipolar electrograms showed a negative T wave (Fig. 1B). On average, the transmural dispersion (maximum difference) in AT along the needles was 8 ± 4 msec. Figure 1C summarizes the data in five hearts. The Epi repolarized slightly, but not significantly, earlier than the Endo (270 ± 20 vs. 266 ± 13, respectively, *P* = 0.5). Also, the difference in ARI duration between the Endo and the Epi (246 ± 13 msec vs. 240 ± 17 msec, respectively) was not statistically significant (*P* = 0.6).

### Electrical measurements in the coronary‐perfused wedge preparation

**Figure 2:**
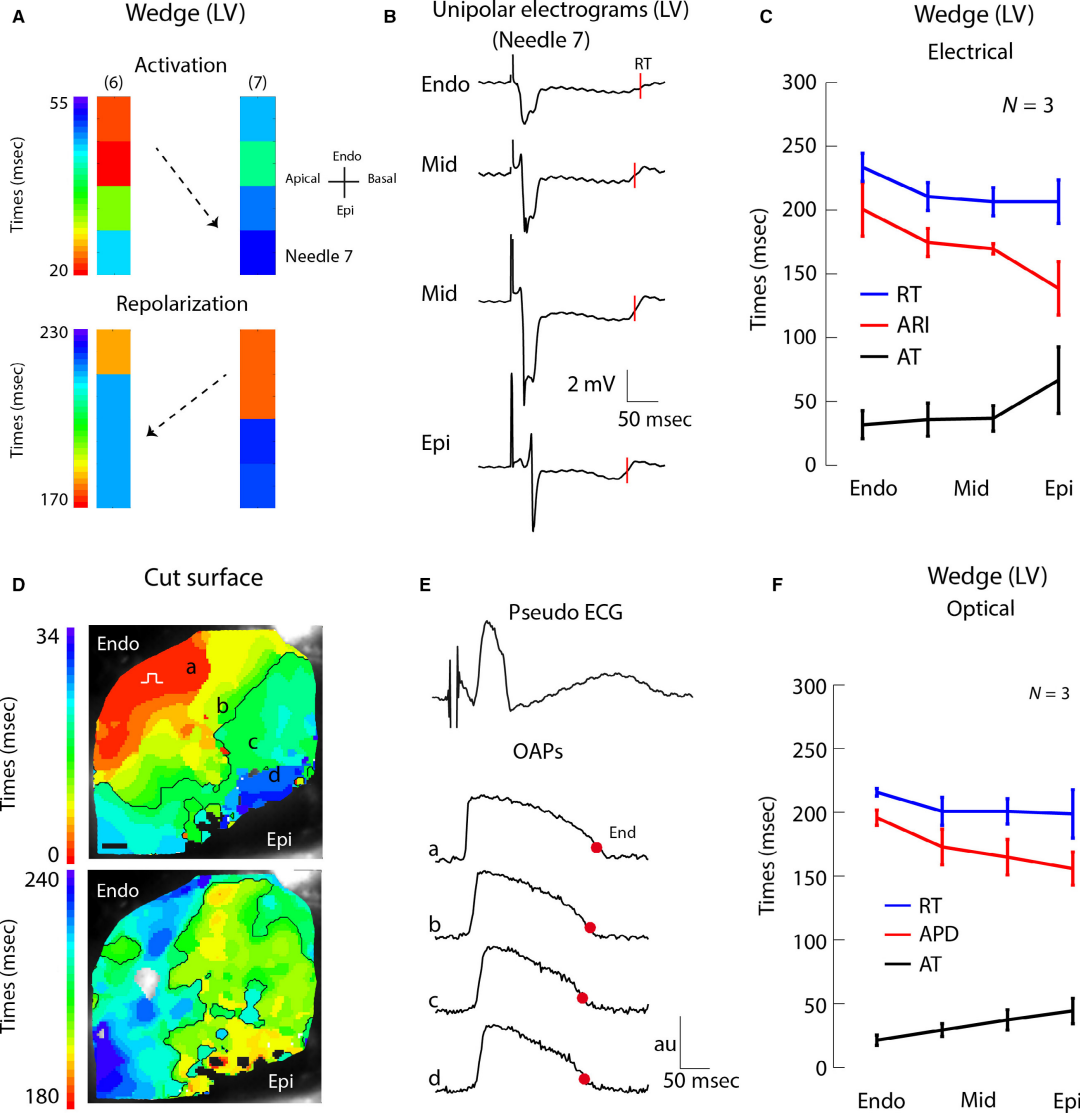
Activation and repolarization sequence in the perfused LV wedge preparation obtained from electrical (A–C) and optical (D–F) recordings. (A) Reconstructed activation (upper) and repolarization (lower) pattern in the LV wedge preparation based on two transmural needle electrodes (pacing interval 800 msec). Black dots represent electrodes. Areas between the black dots were interpolated. (B) Unipolar electrograms recorded with needle 7 (same needle as in Fig. 1B). The red lines indicate the moments of repolarization. (C) The line graph shows the average activation and repolarization times and ARI gradient along needle 7 in three animals. (D) Reconstructed activation (upper) and repolarization (lower) patterns in the LV wedge preparation based on optically recorded action potentials. (E) The upper tracing shows a pseudo‐ECG recorded simultaneously with the optical potentials. The three lower tracings show optical action potentials at regions indicated in panel (D) The red dots represent the 80% of repolarization. (F) The line graph shows the average activation and repolarization pattern and APD gradient at the position of needle 7 in three animals. Au, arbitrary units; LV, left ventricle; AT, activation time; RT, repolarization time; Endo, subendocardium; Mid, mid‐myocardium; Epi, subepicardium; OAP, optical action potentials; APD, action potential duration; ECG, electrocardiogram.

LV wedge preparations were created from all five hearts. Analysis, however, was done only on three preparations since two preparations were excluded because of poor signal‐to‐noise ratio. Figure S1 A shows average AT, ARI, and RT values for the remaining three animals in the intact heart. Figure 2A shows the activation and repolarization patterns based on two needles (eight electrodes) located in the middle of the wedge preparation (called internal in the remainder of the manuscript), remote from the cut surface. In one dog, the electrograms showed ST‐segment depression; whereas in the other two dogs, the electrograms had a clear iso‐electric ST segment. To determine whether transmural gradients differed between activations, six subsequent complexes in each wedge were analyzed. The difference in ARI between activations was 6 ± 0.8 msec and variability in ARI was 4 ± 0.8 msec (Fig. S1B). Transmural gradients were similar for every activation in all wedge preparations (53 ± 18 msec, *n* = 3). Figure 2B displays the electrograms measured with the same needle, and from the same dog, as in Figure 1B. Figure 2C shows the average AT, RT, and ARIs along that needle in three dogs (for the intact heart data, see Fig. S1). In those preparations, transmural activation took 35 ± 15 msec. The Epi repolarized earlier, although not significantly, than the Endo (207 ± 16 vs. 234 ± 11 msec, respectively, *P* = 0.35). In one preparation, there was no transmural difference in ARI; whereas in two preparations, the ARI was approximately 60 msec shorter at the Epi than in the Endo (Fig. S2). On average, the ARI was 202 ± 21 ms in the Endo and 139 ± 21 msec in the Epi (*P* = 0.19) at a pacing cycle length of 800 msec.

To investigate whether the ARI gradients at the cut surface of the wedge preparation were representative for the internal wedge gradients, the ARIs measured in the internal wedge were correlated with those recorded at the cut surface. One needle was left positioned at the border of the perfused area as the wedge preparation was dissected in order to make recordings of the cut surface. Unfortunately, in one preparation, the needle was outside the perfused area and had to be replaced after the wedge was created, which led to an unhealed injury potential. The latter made it impossible to determine the AT, RT, and ARI. In the other two preparations, local electrograms with ST‐segment depression were measured (Fig. S3). The ARI calculated from those electrograms correlated significantly with those from the middle of the wedge (*r* = 0.97, slope=1,1, *P* = 0.00005), indicating that, despite the ST‐segment depression pointing to remote ischemia, the ARI recorded internally represented the ARI measured at the cut surface.

### Optical measurements in the perfused wedge preparation

Activation and repolarization patterns were then analyzed at the cut surface of the wedge preparation with optical mapping. Figure 2D–E shows the reconstructed activation (upper) and repolarization pattern (lower) based on optically recorded potentials. The transmural activation time was 23 ± 6 msec (Fig. 2F). The repolarization times in the Endo and Epi (215 ± 3 msec vs. 198 ± 19 msec, respectively, *P* = 0.67) were not different, similar to the patterns measured with the needle electrodes in the wedge preparation. The APD on the cut surface of the wedge preparation, however, was significantly longer in the Endo than in the Epi (difference of 34 ± 22 msec, *P* = 0.003).

**Figure 3:**
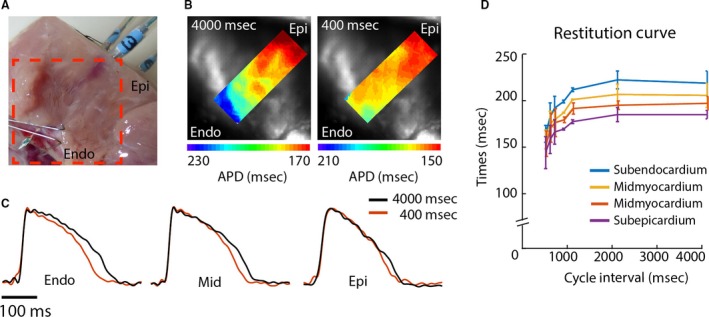
Action potential restitution characteristics at the cut surface of the coronary‐perfused LV wedge preparation. (A) Photograph of the LV wedge preparation showing the field of view used for the optical recordings. (B) Reconstructed action potential duration gradients near the contact region of the needle electrode during 4000 msec (left) and 400 msec (right) stimulus intervals. (C) Optical action potentials at the subendocardium, mid‐myocardium, and subepicardium during 4000 msec (black) and 400 msec (red) stimulus intervals. (D) The line graph shows the average APD at different cycle lengths in the subendocardium (Endo), mid‐myocardium (Mid), and subepicardium (Epi). Au, arbitrary units.

We did not observe Mid cells with long APDs (M cells) in any of the preparations with either high‐resolution optical mapping or needle electrodes. In addition, at the longest pacing cycle length (4000 msec), APDs in the Mid were between those at the Endo and Epi (Fig. 3).

### The relation between action potential durations (optical) and ARIs (electrical) in the wedge preparation

**Figure 4:**
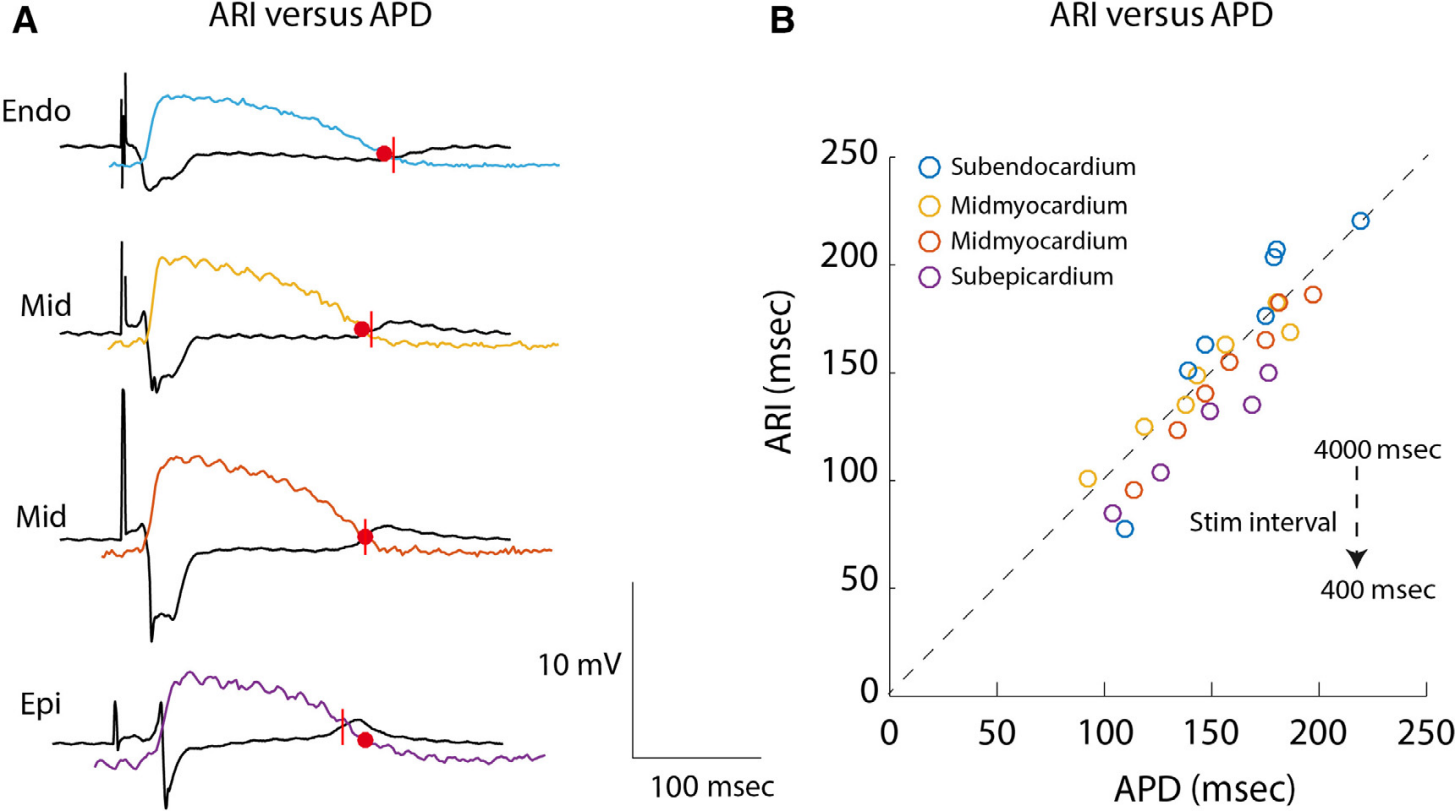
The relation between electrically recorded activation recovery intervals and optically recorded action potentials. (A) The tracings show transmurally recorded electrograms from the internal wedge preparation and superimposed optical action potentials recorded at the cut surface. (B) The graph shows the correlation between optically recorded action potential durations and activation recovery intervals recorded from the internal wedge preparation from the subendocardium, mid‐myocardium, and subepicardium at decreasing cycle intervals. Endo, subendocardium; Epi, subepicardium; Mid, mid‐myocardium; APD, action potential duration; ARI, activation recovery interval.

Figure 4A displays optical recordings at the cut surface of the wedge preparation superimposed on the simultaneously recorded unipolar electrogram of the needle electrode in the center of the wedge preparation. The decision to correlate the APD from the cut surface with the ARI measured at the internal site (4 mm distance) was made because ST‐segment depression was present at the cut surface. Across all three wedge preparations, the ARI duration at the internal site correlated with the APD at the cut surface (*R* = 0.68, slope = 0.51, *P* = 0.016). If the optical and electrical signals were aligned at the moment of activation, then the end of the action potential (red dots) corresponded to the rising phase of the T wave in the local electrograms (red lines) (*R* = 0.67, slope = 0.85, *P* = 0.019). Accordingly, restitution characteristics based on optical APD and electrical ARIs did not differ (Fig. S3C). Furthermore, the correlation between the ARI and APD remained significant in all myocardial regions when cycle length was reduced from 4000 msec to 400 msec (Endo *R* = 0.95, slope 1.3, *P* = 0.001, Mid1 *R* = 0.98, slope 1.10, *P* = 0.0001, Mid2 *R* = 1.00, slope = 1.00, *P* = 0.00001, Epi R = 0.98, slope = 1.1, *P* = 0.00009, Fig. 4B).

### The relation between ARIs measured in the intact heart and in the wedge preparation

**Figure 5:**
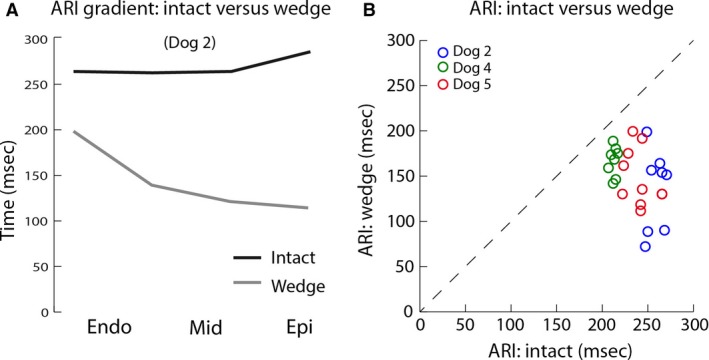
The relation between activation recovery intervals recorded from the intact heart and LV wedge. (A) The graph shows the activation recovery interval gradients in the intact heart and the LV wedge at the same location during 800 msec cycle intervals. (B) The graph shows the correlation between activation recovery intervals recorded in the intact heart and the LV wedge at the same location in three animals during pacing with 800 msec cycle interval. Endo, subendocardium; Epi, subepicardium; Mid, mid‐myocardium; ARI, activation recovery interval.

To test whether the results obtained from the wedge preparations could be translated to the intact heart, the ARIs were compared. Figure 5A shows that at pacing cycle length 800 msec, there was a clear ARI gradient in the wedge preparation but not in the intact heart of animal 5. That phenomenon was observed in all three animals (Fig. S2). Consequently, the ARI in the wedge preparation did not correlate with the ARI in the intact heart (Fig. 5B). On average, the ARI was shorter in the wedge preparation than in the intact heart (157 ± 10 vs. 238 ± 14 msec, *P* = 0. 003). Furthermore, the transmural gradients in AT, ARI, and RT were larger in the wedge preparation than in the intact heart (Table 1).

**Table 1:** Transmural gradients in the Langendorff‐perfused intact heart and wedge preparation

	Intact heart	LV wedge preparation	
	Electrical	Electrical	Optical
AT (msec)	8 ± 4^a^	35 ± 15^b^	23 ± 6^b^
ARI/APD (msec)	5 ± 16^a^	−62 ± 32	−40 ± 10^b^
RT (msec)	13 ± 7^a^	−27 ± 22	−17 ± 13

ARI, activation recovery interval; AT, activation times; LV, left ventricular.

a Means significantly shorter than in the LV wedge preparation (*P* < 0.05), *n* = 3.

b Means significantly different from zero (*P* < 0.05), *n* = 3.

## Discussion

Data obtained in this study indicated that: (1) the ARI calculated from unipolar electrograms recorded in the wedge preparation represented local action potential duration; and (2) the ARI recorded in the wedge preparation did *not* correlate with the ARI measured in the intact heart using plunge needle electrodes. Gradients of ARI were larger in wedge than in the intact Langendorff‐perfused heart. Thus, discrepancies between results obtained in wedge preparations and intact hearts are not due to methodology but caused by differences between the two preparations.

This study addressed whether optically recorded transmembrane potentials recorded from the LV wedge preparation correlated with the extracellular potentials recorded with unipolar electrodes. The relation between transmembrane potentials and electrically recorded extracellular potentials was thoroughly addressed by Spach et al. (1972, 1979) in the 1970s. The maximum negative dV/dt of the local electrogram has been shown to coincide with the maximum positive dV/dt of the upstroke of the action potential (Dower 1962). The maximum negative dV/dt of the transmembrane potential also has been shown to correlate with the maximum positive dV/dt of the T wave of the local electrogram (Potse et al. 2009). Those relationships should also occur with optically recorded action potentials (Efimov et al. 1994). However, the repolarization phase of the OAP recorded from tissue is slow, leading to a low maximum negative dV/dt that is often undetectable due to noise. In that setting, it has been found to be more accurate to determine the end of the OAP at 80% of repolarization (Potse et al. 2009). The data in this study showed that the correlation between the ADP80 of the optically recorded action potentials and ARIs measured with needle electrodes was significant and indicative of a similar determination of repolarization.

This study also compared unipolar electrograms recorded from Langendorff‐perfused intact hearts with those recorded at the same location in a LV wedge preparation derived from the same heart. A transmural gradient in ARI or repolarization was not found in the intact heart, similar to previous findings by others during Langendorff perfusion (van Dam and Durrer 1961) or in open‐chest canines (Anyukhovsky et al. 1996; Opthof et al. 2007b; Voss et al. 2009). ARIs in this study were shorter in the LV wedge preparation than in the intact heart; moreover, the wedge preparation showed a clear transmural gradient. Consequently, the ARIs in the LV wedge preparation did not correlate with the ARIs in the intact heart, demonstrating the existence of large electrophysiological differences between the LV wedge preparation and the intact heart. Discrepancies between ARI gradients in the intact canine heart and its derived LV wedge preparations have been reported previously (Anyukhovsky et al. 1996; Voss et al. 2009). A comparison of studies performed in the intact heart showed that the absence of a transmural gradient in ARI or repolarization times was a consistent finding (Anyukhovsky et al. 1996; el‐Sherif et al. 1996; Opthof et al. 2007b; Voss et al. 2009). However, results derived from wedge preparations were less consistent. The majority of studies in the canine LV wedge preparation reported M cells (Sicouri and Antzelevitch 1991; Yan et al. 1998; Akar et al. 2002). M cells were not observed in the preparations of this study. Both this study and previous studies in the canine LV wedge preparation showed longer APD in the Endo than in the Epi (Shimizu and Antzelevitch 1998; Akar et al. 2002; Poelzing and Rosenbaum 2004). Other studies either did not find a transmural gradient in ARI (Voss et al. 2009; Janse et al. 2012) in the LV wedge preparation or a longer APD in the Epi than in the Endo (Anyukhovsky et al. 1996).

The large variability in the results obtained in LV wedge preparations suggests that it is more susceptible to artifacts induced by dissection, handling, or other experimental conditions compared to the intact heart preparation. For example, an incision perpendicular to the fiber direction could result in electrotonic uncoupling which would lead to M cell‐like behavior, which allegedly is masked in the intact myocardium (Conrath et al. 2004). Interestingly, M cells seem to appear after perpendicular, but not fascicular, incisions (Janse et al. 2012). Thus, M cells may show up at the cut surface, but not in the internal zone of a wedge preparation. Needle electrodes perforate the myocardium and generate a cut surface as well, but are required for transmural recording of activation and repolarization. We did not observe long ARIs, indicative for M cells, at the cut surface or in the internal zone of the wedge preparation. The transmural ARI gradient at the cut surface correlated significantly with the transmural ARI gradient in the wedge preparation. Alternative explanations for M‐cell behavior could be internalization or downregulation of I_Ks_ and I_Kr_ channels during the time course of the experiment or local prolongation of APD by Purkinje muscle junctions (Ng et al. 2014; Walton et al. 2014). The latter would make the observation of M cells a matter of chance, since not every plane within the transmural wall includes these junctions, which would explain why M cells are reported in one study in the canine wedge, but not in the other.

### Study limitations

In this study, intact hearts were measured during Langendorff perfusion with a mixture of blood and Tyrode's solution; whereas LV wedges were perfused with Tyrode's solution. The latter should not have affected APD (Rosen et al. 1972). However, whether Tyrode's solution provides sufficient oxygen to myocardium is a debated issue (Kuzmiak‐Glancy et al. 2015). Anoxia shortens the action potential and could affect transmural gradients in ARI or APD (Morena et al. 1980). However, the QT times recorded from LV wedge preparations in this study averaged 284 ± 13 msec and were not different from previous publications on similar‐sized LV wedge preparations. This would indicate that the preparations in this study were not anoxic (Pajouh et al. 2005; Di Diego et al. 2013). The insertion of the needle electrodes caused varying degree of damage in one wedge preparation but not in the others. This argues against chronic lesion caused by the needles. In addition, the same needles were used for each experiment, which makes it also unlikely that the needles caused chronic lesion. Another difference between the intact and the wedge preparation is the use of the excitation–contraction uncoupler blebbistatin to facilitate optical recordings. Mechanical contraction in these experiments was always triggered from endocardium to epicardium, and therefore could alter morphology of T waves of electrograms differently at the endocardium versus epicardium. Thus, contractions could affect transmural gradients in ARI and contribute to the observed difference in ARI gradients between intact heart and wedge preparations (Opthof et al. 2015). In addition, the effect of blebbistatin on electrophysiological parameters has been debated (Lou et al. 2012; Brack et al. 2013). However, this study assumes that any effect of blebbistatin would affect the preparation homogeneously and therefore not alter any gradients in ARI or APD.

## Conclusion

Our study shows that APD patterns in the LV wedge preparation do not resemble those present in the intact heart. It may be that an explanation for this observation can be found in the different experimental conditions in which the preparations have been measured. However, many studies have measured wedge preparation in similar experimental conditions as we have and drawn conclusions that were extrapolated to the intact heart. Our data suggest that such extrapolation should not be made. However, the wedge preparation remains a suitable tissue model for studying arrhythmogenesis.

## Conflict of Interest

The authors have no competing interest to declare.

## Supporting information




**Figure S1.** Activation and repolarization sequences in the left ventricle of a Langendorff‐perfused canine heart.
**Figure S2.** Overview of the transmural gradient in activation recovery interval in each dog.
**Figure S3.** Comparison between activation recovery interval at the cut surface and the internal wedge.
