# Supplementary material for: Local transmural action potential gradients are absent in the isolated, intact dog heart but present in the corresponding coronary‐perfused wedge
Source: Physiol Rep. 2017 May 29;5(10):e13251. doi: 10.14814/phy2.13251 (PMC5449556; doi:10.14814/phy2.13251)

Supplemental Figure 1

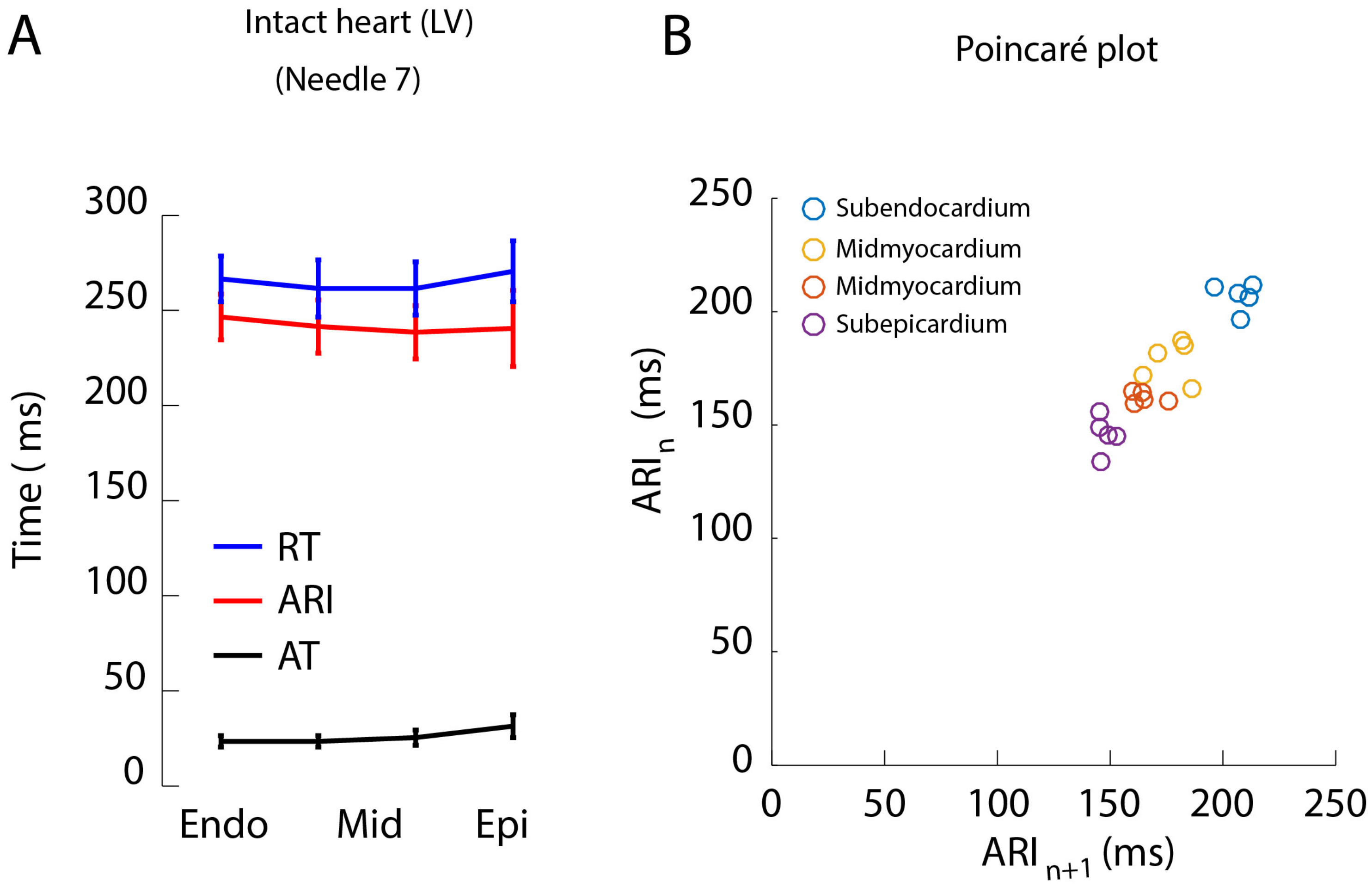

Supplemental Figure 2

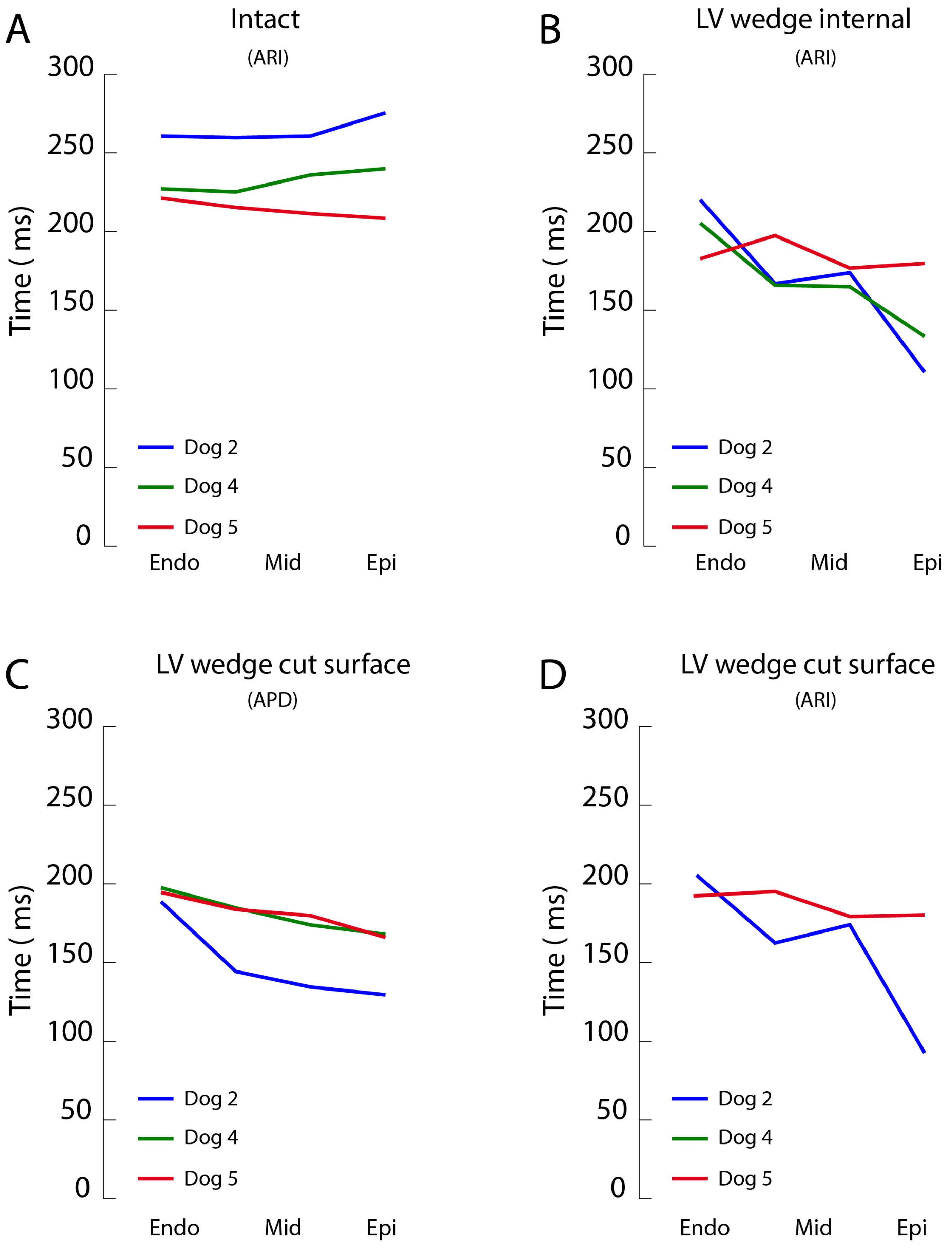

Supplemental Figure 3

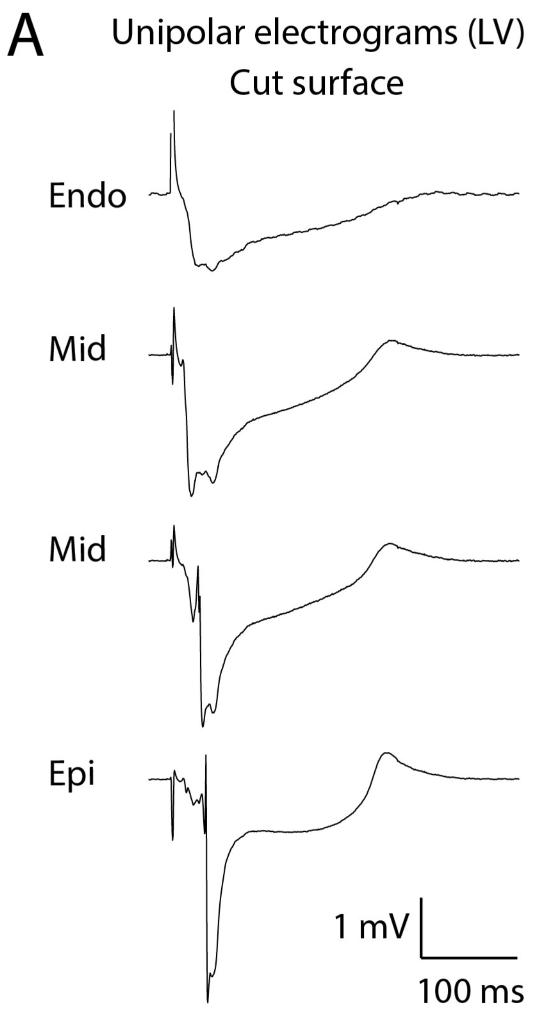

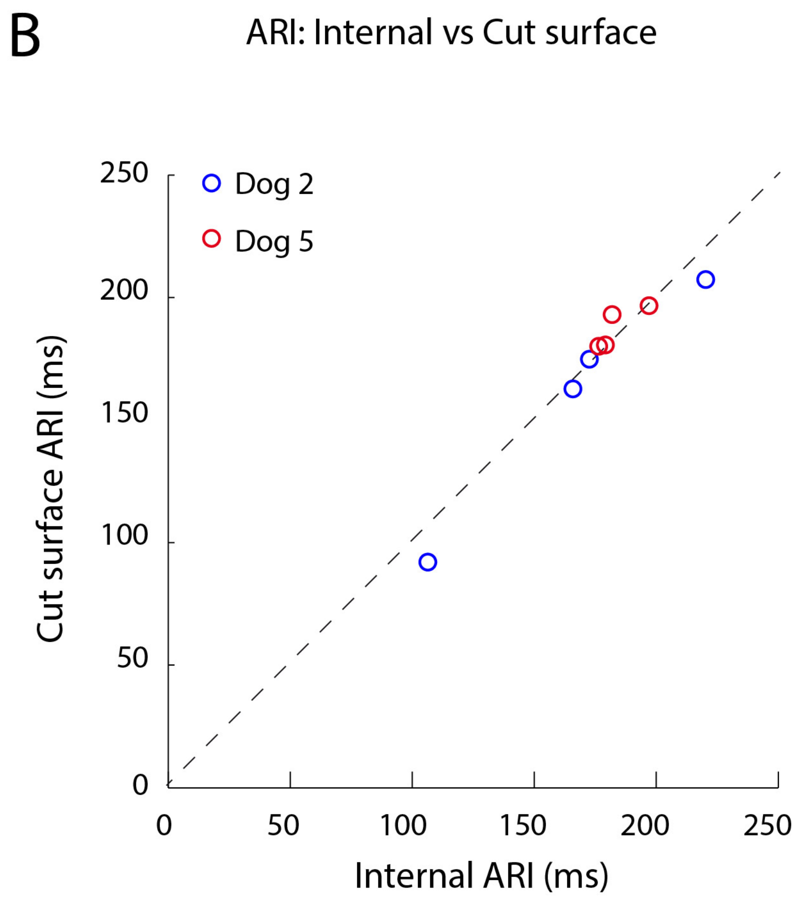

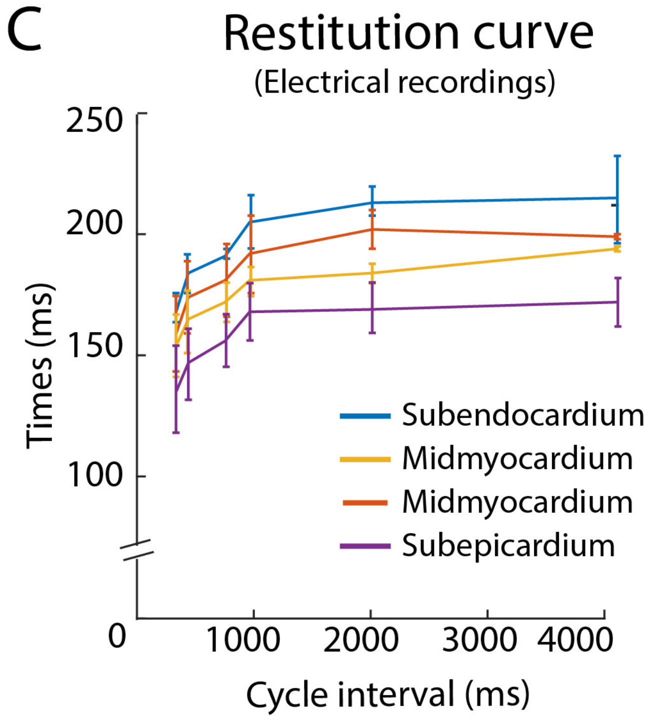

Supplement: Supplementary file 1 — Figure S1. Activation and repolarization sequences in the left ventricle of a Langendorff‐perfused canine heart. Figure S2. Overview of the transmural gradient in activation recovery interval in each dog. Figure S3. Comparison between activation recovery interval at the cut surface and the internal wedge. [file PHY2-5-e13251-s001.pdf]
